# Case Report: A new therapeutic option for a rare patent ductus arteriosus child complicated with pulmonary artery aneurysm due to infectious endocarditis

**DOI:** 10.3389/fcvm.2025.1493730

**Published:** 2025-02-19

**Authors:** Hua-yong Zhang, Xiao-yuan Feng, Chang-jian Li, Yong Zhang

**Affiliations:** ^1^ Department of Cardiology, Wuhan Children’s Hospital (Wuhan Maternal and Child Healthcare Hospital), Tongji Medical College, Huazhong University of Science & Technology, Wuhan, China; ^2^ Clinical Medical Research Center for Birth Defect Prevention and Treatment in Wuhan, Wuhan, China; ^3^ Department of Cardiac Ultrasonography, Wuhan Children’s Hospital (Wuhan Maternal and Child Healthcare Hospital), Tongji Medical College, Huazhong University of Science and Technology, Wuhan, China

**Keywords:** infective endocarditis, patent ductus arteriosus, pulmonary artery aneurysm, children, new therapeutic option

## Abstract

Patent ductus arteriosus (PDA) is one of the most common forms of congenital heart disease (CHD). Infectious endocarditis (IE) is a rare but serious complication of PDA. PDA combined with pulmonary artery aneurysm (PAA) due to IE is rare in children. In this report, we report a rare pediatric PDA case, complicated with PAA due to IE. Transcatheter closure of PDA without the surgical intervention of PAA was performed, with a favorable prognosis.

## Introduction

Patent ductus arteriosus (PDA) accounts for approximately 10% of congenital heart disease (CHD) and is present in approximately 2–4 of every 1,000 births. It is a life-threatening risk factor in prematurely delivered babies, with a higher morbidity (1–3). Infective endocarditis (IE) is undoubtedly one of the most serious complications of CHD (4, 5). IE is frequently caused by Staphylococcus, Streptococcus, and Enterococcus bacteria, etc., among which Staphylococcus aureus is considered to be the most common pathogen (6, 7). What's more, it is particularly worth mentioning that CHD is the leading predisposing factor for IE in children. *Staphylococcus aureus* and *Streptococcus viridans* are the most common pathogenic microorganisms for pediatric IE (8). Intracardiac vegetation is a typical clinical manifestation of IE and is composed of large numbers of platelets, fibrin, pathogenic bacteria, and inflammatory cells. The special structure of infective vegetation facilitates bacterial evasion of the immune system and hinders antimicrobial action, resulting in suboptimal treatment effectiveness (1, 8). Pulmonary artery aneurysm (PAA) is a rare clinical disease with an incidence of about 1 case/14,000 individuals in necropsies (9). According to the pathogenesis, PAA is classified as either congenital or acquired PAA. Congenital PAA can be caused by CHD, congenital pulmonary valve disease, connective tissue disease, etc. Acquired PAA is often secondary to pulmonary hypertension, vasculitis, infectious diseases, malignant neoplastic diseases, medical or traumatic injuries, etc. (10, 11). The management of acquired PAA is still controversial in the current study, and the choice of conservative or surgical intervention varies according to individual differences (9–14). For PDA cases with PAA due to IE, some scholars have opted for the more aggressive treatment of pulmonary artery reconstruction under extracorporeal circulation (11, 13). However, it is worthwhile to discuss whether a conservative strategy can be adopted for children with long duration of small PAA, which is not combined with PAA dissection or rupture. Therefore, we report a rare pediatric PDA case, complicated with PAA due to IE, and share the experience of management from our center.

## Case presentation

**Figure 1:**
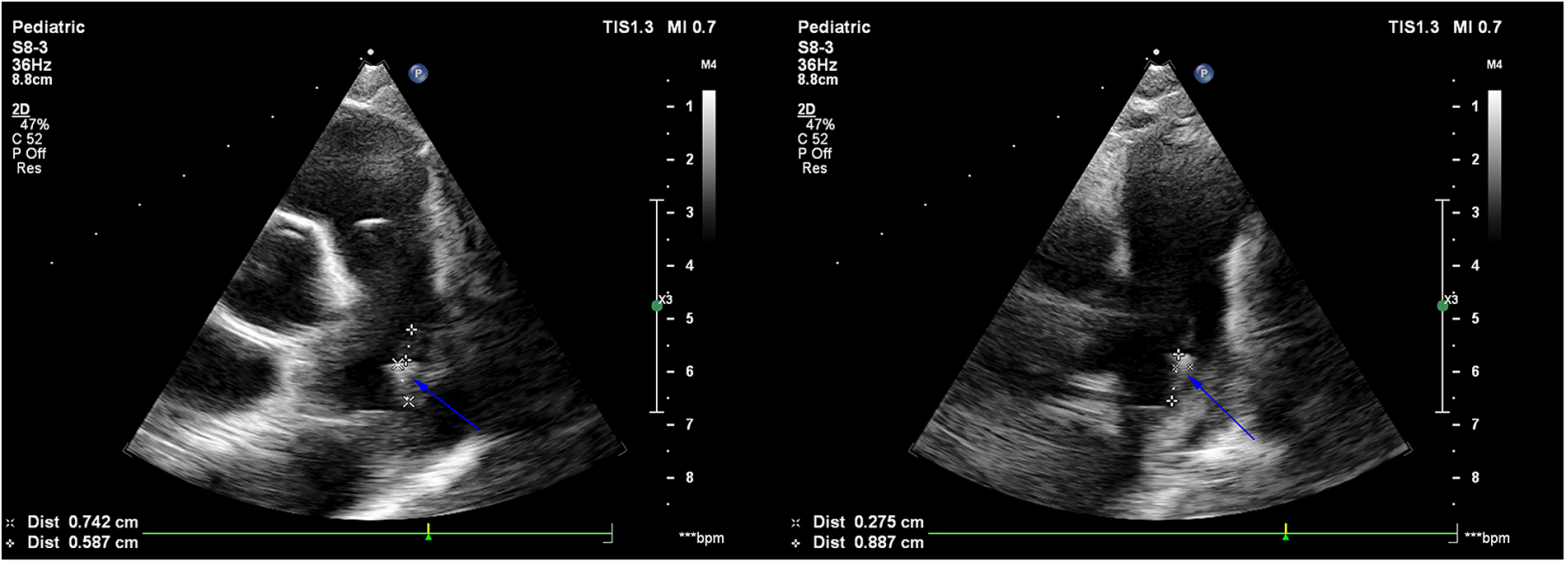
Echocardiogram showed a strip-shaped echo of about 12 mm × 3 mm in size found at the beginning of the left pulmonary artery on the distal left wall of the pulmonary artery, which was fixed in the base, softer in the middle and lower parts, and oscillating obviously, which was considered to be infective vegetation (blue arrow).

A 7-year-old girl, with a 3-year history of heart murmur was admitted to our hospital in July 2024. The girl was admitted to our hospital 3 years ago due to persistent fever for 7 days, during which a heart murmur was detected on routine auscultation, and an Osler nodule was noted in the left thumb. Echocardiogram showed PDA, and strip-shaped infective vegetation of about 12 mm × 3 mm in size (Figure 1). What's more, two blood cultures taken 1 day apart suggest *methicillin-resistant Staphylococcus aureus* (MRSA). Therefore, IE, as well as MRSA sepsis were diagnosed clearly. The anti-infective treatment strategy with vancomycin combined with rifampicin was administered over 6 weeks, according to the drug sensitivity test results. Subsequently, three follow-up blood cultures were negative, while multiple follow-up echocardiograms suggested shrinkage of the infective vegetation. Following multidisciplinary discussion, further surgical intervention was judged unnecessary, so the child was discharged and subsequently discharged to regular outpatient follow-ups. During the follow-up period, a repeat echocardiogram showed resolution of the infective vegetation at a 6-month follow-up. Unfortunately, until this admission, the girl did not receive regular outpatient follow-ups and further treatment for PDA due to socioeconomic factors and personal reasons.

**Figure 2:**
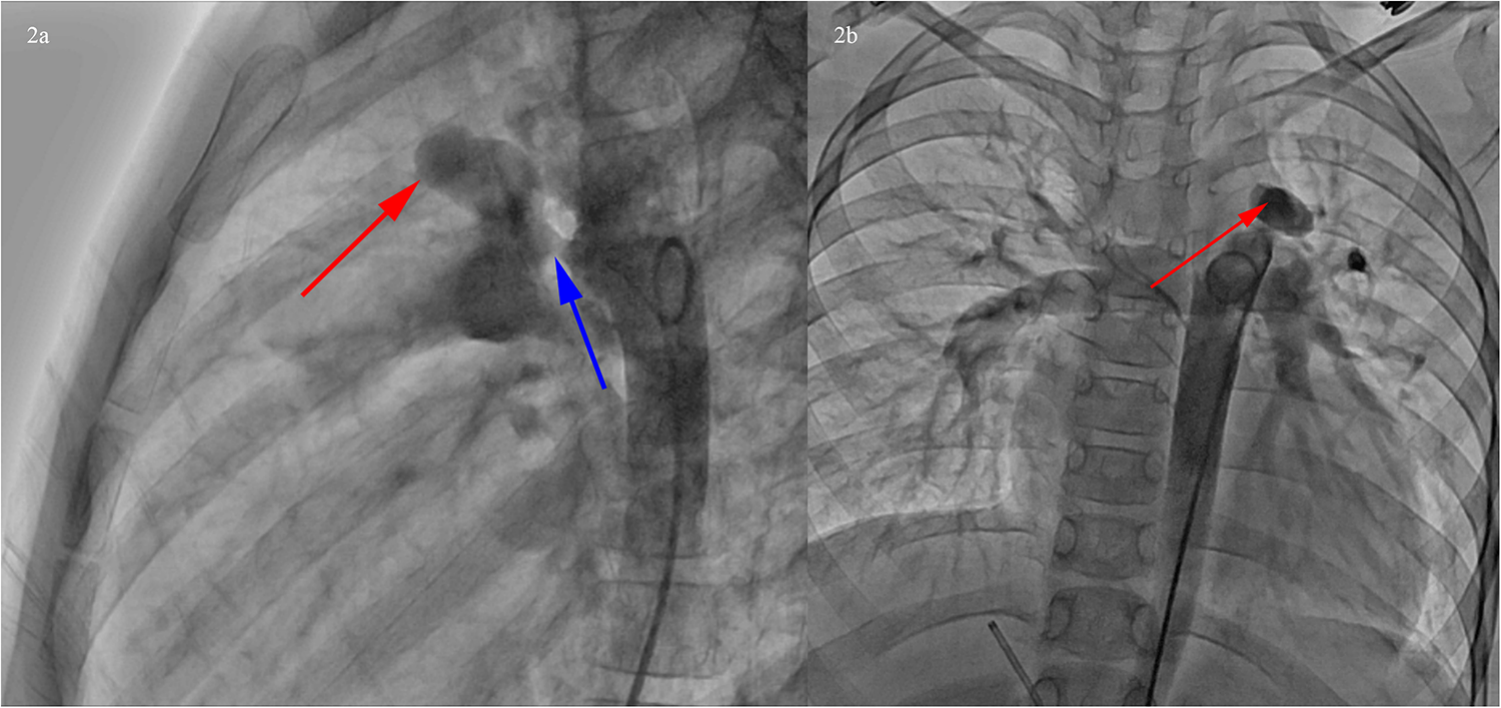
Cardiac catheterization showed PDA (funnel-shaped, narrowest width of 5 mm), and PAA at the junction of the main pulmonary artery and the left pulmonary artery. **(a)** Left lateral position, PDA (blue arrow), PAA at the junction of the main pulmonary artery and the left pulmonary artery (red arrow). **(b)** Posteroanterior position, PAA at the junction of the main pulmonary artery and the left pulmonary artery (red arrow).

Transcatheter occlusion of PDA was proposed on this admission. The preoperative Echocardiogram showed PDA without other abnormal ultrasound signs. The cardiac catheterization showed PDA (funnel-shaped, narrowest width of 5 mm), and PAA at the junction of the main pulmonary artery and the left pulmonary artery (Figure 2).

**Figure 3:**
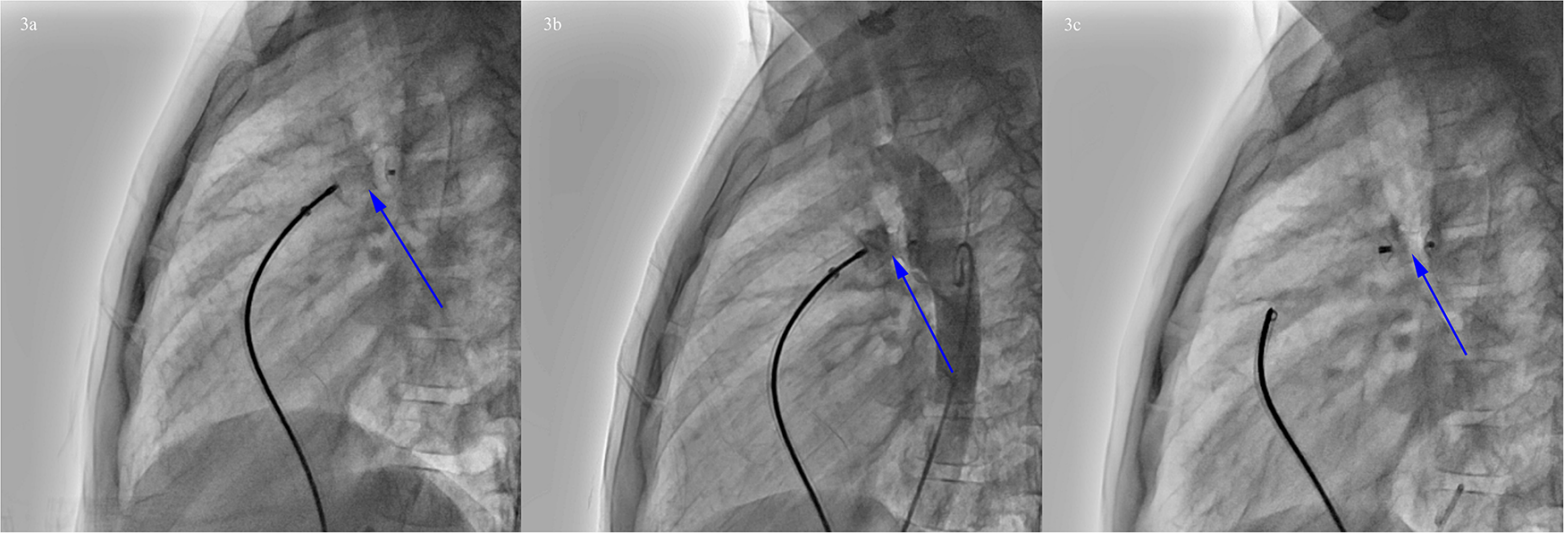
Procedure of PDA occlusion. **(a)** Transcatheter closure of PDA. **(b)** Angiography showed that the occluder was in a good position without residual shunt. **(c)** Rotating the cable to release the occluder (blue arrow).

Based on the results of cardiac catheterization, transcatheter closure of PDA using a 14 mm PDA occluder (*ShangHai Shape Memory Alloy, Shanghai, China*) was performed (Figure 3). The operation went well without any complications. The echocardiogram showed that the occluder was in a good position without residual shunt.

**Figure 4:**
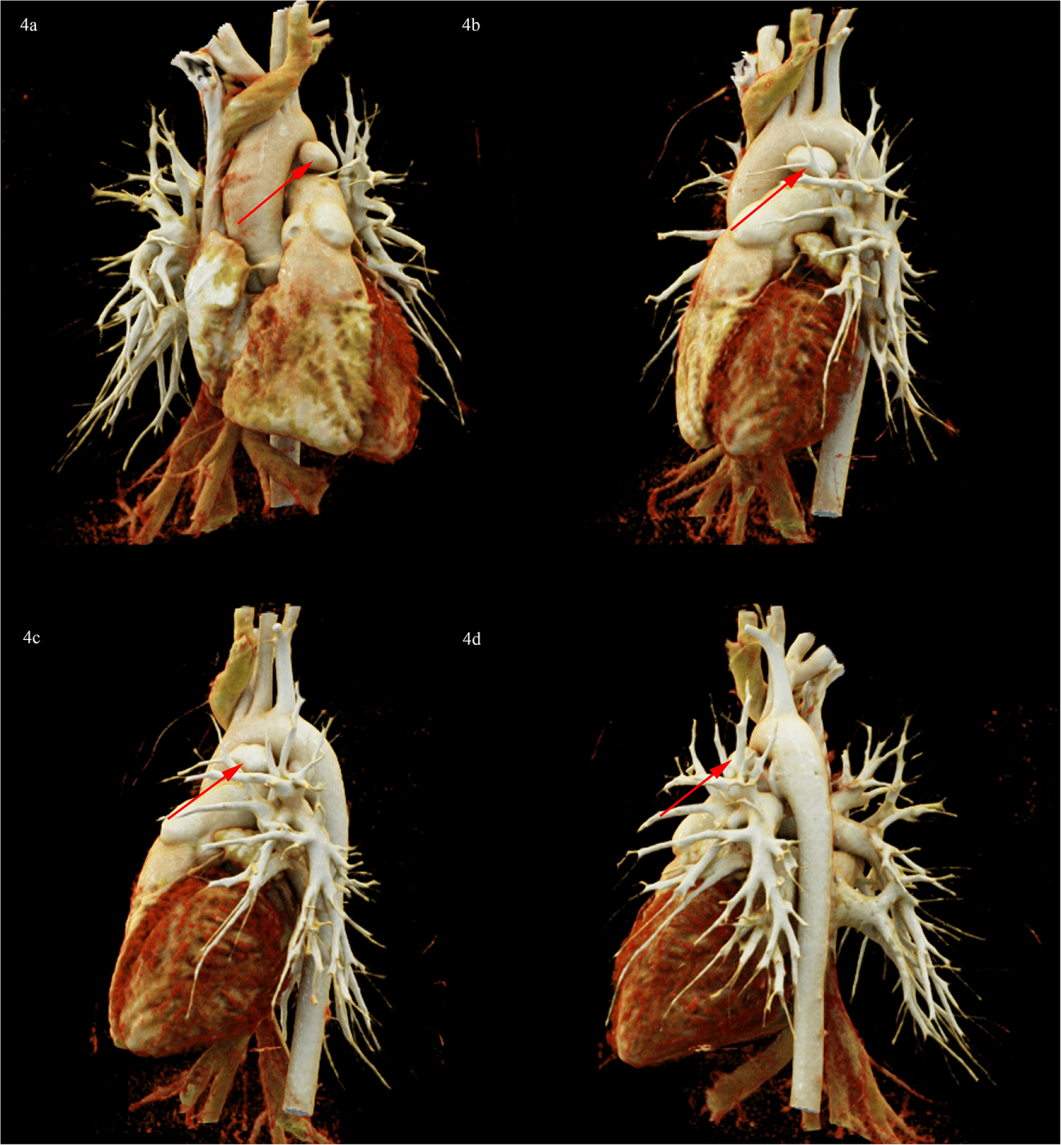
CCTA 3D reconstruction diagram showed PAA at the junction of the main pulmonary artery and the left pulmonary artery, ranging from approximately 10 mm × 13 mm × 18 mm (red arrow). **(a)** Posteroanterior position. **(b)** Left anterior oblique position. **(c)** Left lateral position. **(d)** Left posterior oblique position.

To clarify the three-dimensional (3D) structure of the PAA. Cardiac computed tomography angiography (CCTA) was detected, suggesting PAA at the junction of the main pulmonary artery and the left pulmonary artery, ranging from approximately 10 mm × 13 mm × 18 mm, without PAA dissection or rupture (Figure 4). following multidisciplinary consultation, it was concluded that the PAA did not require further intervention for the time being, with regular outpatient follow-up. The reasons are as follows: (i) PAA with a small diameter of less than 2 cm, without dissection or rupture; (ii) PDA has been successfully occluded without an aorto-pulmonary shunt and with normal pulmonary artery pressure; (iii) The long history of 3 years of disease, with a lower risk of PAA dissection and rupture.

## Discussion

In this study, we report a rare pediatric PDA case, complicated with PAA due to IE. PDA with IE is a relatively rare but serious clinical condition, with an incidence of approximately 0.65% reported in the literature (4). The development of IE may be associated with prolonged impingement of a high-speed aorto-pulmonary shunt due to PDA. Sustained, high-velocity aorto-pulmonary shunts have the potential to inflict endovascular damage and collagen exposure in the pulmonary arteries, thereby increasing the likelihood of IE (4, 15). In cases of PDA combined with IE, the formation of infective vegetation is typically observed in the proximal part of the left pulmonary artery and the bifurcation of the pulmonary artery (16, 17). According to the drug sensitivity test results, the case was discharged from the hospital with clinical improvement after 6 weeks of active anti-infective treatment, and infective vegetation disappeared on repeat echocardiography at 6 months, suggesting a good clinical outcome. In addition, due to the limitations of echocardiography, focal PAA and PAA dissection may be detected at a low rate. CCTA should be performed during hospitalization and follow-up in children with infective pulmonary artery vegetation to avoid missed or delayed diagnosis and treatment.

PAA diagnosis is controversial, with some scholars suggesting that pulmonary artery dilation greater than 1.5 times the upper limit of normal is diagnostic (9). CHD in combination with Pulmonary hypertension is a common cause of pulmonary artery dilatation. The pathogenesis is related to abnormal pulmonary hemodynamics (18). In cases of pulmonary artery dilatation, approximately 5.5% meet diagnostic criteria for PAA (19). PAA due to CHD is referred to as true PAA, and the mechanism of their formation may be related to sustained pressure and volume overload involving the entire layer of the pulmonary artery vessel wall (9). Among CHD, PAA is most common in PDA and pulmonary stenosis (19). The child in this study had never had the cardiac malformation corrected for socioeconomic factors and personal reasons, resulting in the persistence of an aortopulmonary shunt, and the continued high pressure and volume overload were the basis for the child to develop PAA. In addition, intimal injury of the pulmonary artery wall and necrosis of elastic fibers of the medial membrane due to IE are also important pathophysiological mechanisms of PAA development (9). In this case, PAA due to the combination of infectious immune damage and prolonged high-velocity blood shocks was found on cardiac catheterization 3 years after the onset of IE. The pathophysiologic evolution of PAA in pediatric cases is rare. Whether PAA requires further intervention is controversial in the current study. Overall, a comprehensive decision needs to be made based on the etiology of the PAA, its hemodynamic impact, and comorbidities (9). Some scholars believe that PAA due to CHD, such as PDA, usually does not have serious clinical consequences if the heart defect is corrected promptly (20, 21). However, PAA due to PDA may be secondary to dissection or rupture of the PAA, threatening the patient's life, as reported by other investigators. Chest pain, dyspnea, cyanosis, hypotension, even hemoptysis, syncope, and sudden cardiac death are common in these cases (12, 21, 22). For these life-threatening PAA cases, the choice of interventional catheterization or surgical treatment varies depending on the child's condition (9, 11, 13). Given the low incidence of PAA dissections, a conservative approach is often used (20). For small, localized PAA, transcatheter intervention may be preferred. Typically, this involves spring coil embolization or overlay pulmonary stent placement (22, 23). If the PAA is large or in combination with a deformity that requires surgical correction, resection of the PAA and reconstruction of the pulmonary artery may be necessary (13, 19, 24). Due to the rarity of pediatric cases, we lack experience in managing such cases. Considering that the history of PAA in our case may be relatively long, the PAA is small, not associated with serious complications such as dissection or rupture, and the surrounding structure of the PAA is still stable, as well as the absence of clinical symptoms. The risk of PAA dissection is low, according to previous studies (20). After multidisciplinary discussion, it was concluded that after transcatheter PDA occlusion, the aortopulmonary shunt flow disappeared, and the pressure and volume loads were suddenly reduced, which theoretically greatly reduced the chance of the dissection or rupture of PAA. At this stage, performing a resection of the PAA and subsequent pulmonary artery reconstruction, or alternatively, implementing overlay pulmonary stenting, could result in considerable trauma. Additionally, there is a potential risk of inducing pulmonary stenosis post-stenting in a growing child, which may ultimately be more detrimental than beneficial. Therefore, we decided to manage the PAA palliatively with regular outpatient follow-up. In addition, we also follow up dynamically, and further intervention is required in case of further enlargement of the PAA or occurrence of dissection or rupture of PAA (13, 20). After nearly 5 months of follow-up, the prognosis is good, and the PAA remains stable.

## Conclusion

PDA is one of the most common forms of CHD. IE is a rare but serious complication of PDA. In this study, we report a rare case of PDA combined with PAA after IE in a child. The course of this case reminds pediatric cardiologists to pay attention to early diagnosis and treatment of CHD, especially PDA, to avoid serious complications. For PDA cases complicated by PAA due to IE, transcatheter closure of the PDA without surgical intervention of the PAA may be the preferred modality when the PAA has a long history, is small, asymptomatic, and has a lower risk of dissection and rupture.

## Data Availability

The original contributions presented in the study are included in the article/Supplementary Material, further inquiries can be directed to the corresponding author.
